# Incidence, predictors and prognosis of acute kidney injury in nonagenarians: an in-hospital cohort study

**DOI:** 10.1186/s12882-020-1698-y

**Published:** 2020-01-30

**Authors:** Andre Luis Bastos Sousa, Leticia Mascarenhas de Souza, Osvaldino Vieira Santana Filho, Victor Hugo Ferreira e Léda, Paulo Novis Rocha

**Affiliations:** Medical School of Bahia of the Federal University of Bahia, Av. Reitor Miguel Calmon, S/N, Vale do Canela, Salvador, BA 40110-100 Brazil

**Keywords:** Nonagenarians, Acute kidney injury, Hemodialysis, Mortality

## Abstract

**Background:**

Given the aging of the population, nephrologists are ever more frequently assisting nonagenarians with acute kidney injury (AKI). The management of these patients presents unique characteristics, including bioethical dilemmas, such as the utilization of renal replacement therapy (RRT) at this extreme age.

**Methods:**

We conducted a retrospective cohort study at a tertiary hospital. Over a 10-year period, 832 nonagenarians were hospitalized for two or more days. A random sample of 461 patients was obtained; 25 subjects were excluded due to lack of essential data. AKI was defined and staged according to the Kidney Disease Improving Global Outcomes (KDIGO) criteria.

**Results:**

We analyzed data from 436 patients, mean age 93.5 ± 3.3 years, 74.3% female; 76.4% required intensive care unit (ICU). The incidence of AKI was 45%. Length of hospital stay, ICU admission, vasopressors, and mechanical ventilation (MV) were independent predictors of AKI. Overall in-hospital mortality was 43.1%. Mortality was higher in the AKI compared to the no AKI group (66.8% vs. 23.8%, *p* < 0.001). Only 13 patients underwent RRT; all were critically ill, requiring vasopressors and 76.9% in MV. Mortality for this RRT group was 100% but not significantly higher than that observed in 26 non-RRT controls (96.1%, *p* = 1.0) obtained by proportional random sampling, matched by variables related to illness severity. In multivariable analysis, age, Charlson’s score, vasopressors, MV, and AKI – but not RRT – were independent predictors of mortality.

**Conclusions:**

AKI is common in hospitalized nonagenarians and carries a grave prognosis, especially in those who are critically iil. The use of RRT was not able to change the fatal prognosis of this subgroup of patients. Our data may help guide informed decisions about the utility of RRT in this scenario.

## Background

Population aging is a worldwide phenomenon, especially in the group of individuals over 80 years old, which is the fastest growing segment of the population [1, 2]. The very elderly have peculiarities that place them at increased risk for acute kidney injury (AKI). However, the literature on AKI in the very elderly is scarce; when caring for these patients, Nephrologists often have to extrapolate data obtained in studies of younger individuals.

In the present century, AKI is a syndrome of the elderly. In 2005, the Beginning and Ending Supportive Therapy for the Kidney (BEST Kidney), a multinational cohort of nearly 30.000 patients, showed that the mean age of patients with AKI was 67 years old [3]. Epidemiological studies have also shown that the chance of developing AKI increases with advancing age [4, 5], possibly due the morphological and functional changes, intrinsic to the renal aging process [4, 6]. In addition, other factors such as co-existing chronic health conditions, polypharmacy and need for invasive procedures contribute to the increased incidence of AKI in the elderly.

The incidence of AKI in elderly patients ranges from 3.1% [5] to 65.6% [7] depending upon the diagnostic criteria adopted, mean age and clinical severity of the individuals studied. However, few studies have been conducted in the very elderly. Wen et al. studied patients with a mean age of 88.5 years and found an incidence of AKI of 14.8% according to AKIN criteria [8]. A recent study showed that AKI on admission was the strongest predictor of in-hospital mortality in a cohort of 283 nonagenarians hospitalized for more than 1 week [9].

Herein, we aim to assess the incidence, risk factors and prognosis of AKI in a random sample of nonagenarians hospitalized for two or more days.

## Methods

### Ethical approval

The study was approved by our local institutional review board (protocol no. 131506/2016). Given the retrospective nature of the study, we were granted a waiver of informed consent. Therefore, no consent to participate was required.

### Design, site and sample

This retrospective cohort study was conducted at a single tertiary hospital in Salvador-Bahia, Brazil. Over a 10-year period (2006 till 2016), there were 4017 medical encounters with nonagenarians at our institution. We focused on those that were admitted to the hospital for at least 2 days; when there were two or more admissions for the same patient, only the most recent was considered. A total of 832 nonagenarians fullfilled our eligibility criteria. To detect a hypothetical incidence of AKI of 50% with a 95% confidence level and a margin of error of 5% we calculated that we needed a minimum of 384 patients. This number was inflated by 20% to account for possible losses, resulting in a final sample size of 461 patients, which were then randomly selected from the pool of eligible patients. Electronic medical records were then screened and patients were excluded if critical data for the diagnosis of AKI (such as serum creatinine levels) were missing.

### Definitions

AKI was defined and classified according to Kidney Disease Improving Global Outcomes (KDIGO) criteria [10]. KDIGO 1 required an absolute increase in serum creatinine (SCr) by ≥0.3 mg/dl in 48 h or an increase in SCr from 1.5 to 1.9 times the baseline value that was presumed or known to have occurred within the prior 7 days. KDIGO 2 required an increase in SCr from 2.0 to 2.9 times the baseline value. KDIGO 3 required an increase in SCr ≥ 3 times the baseline value; or an increase in SCr to ≥4 md/dl; or onset of renal replacement therapy (RRT) [10]. The codification of AKI in the dataset was made by a nephrologist (ALBS) after careful analysis of the SCr curve for each patient. When there was any doubt about the diagnosis or stage of AKI, a second nephrologist (PNR) was asked to review the case. When there were discordant opinions between the two nephrologists, a consensus was reached. Patients admitted with normal or stable renal function who later met AKI criteria during hospitalization were classified as having hospital-acquired AKI. Patients admitted with a rising SCr or with an elevated SCr which then returned to values less than or equal to 1.2 mg/dl (normal laboratory reference) or had a 50% decrease from the highest SCr value, were classified as having community-acquired AKI. Patients admitted with an elevated SCr that remained stable during the hospital stay were considered to have chronic kidney disease. The urine output criteria of the KDIGO guidelines were not used in this study because this information was missing from the electronic medical records of the majority of patients.

### Statistical analysis

Categorical variables were summarized using simple and relative frequencies and compared between two groups using the Chi-square or Fisher’s tests, as appropriate. Continuous variables were summarized by mean and standard deviation or median and interquartile range, and compared between two groups using Student’s t test. The incidence of AKI was calculated by dividing the number of new cases of AKI by total number of patients exposed to the risk. In order to identify predictors of AKI and in-hospital mortality, we performed univariate and multivariable logistic regression analyses. Variables with *p* values < 0.10 on univariate analyses were selected for inclusion in stepwise backward multivariable logistic regression models. Variables with p values < 0.05 in final analyses were considered statistically significant. Receiver operating characteristic (ROC) curves were constructed to evaluate the predictive / diagnostic ability of the multivariable logistic regression models. To further explore the relationship between RRT and mortality, we created a non-RRT control group (2 controls for 1 case) by proportional random sampling, matched for the variables: AKI, ICU admission, use of vasopressors and mechanical ventilation. Sampling procedures and statistical analyses were performed using the Statistical Package for Social Sciences (SPSS) version 17.0.

## Results

**Fig. 1:**
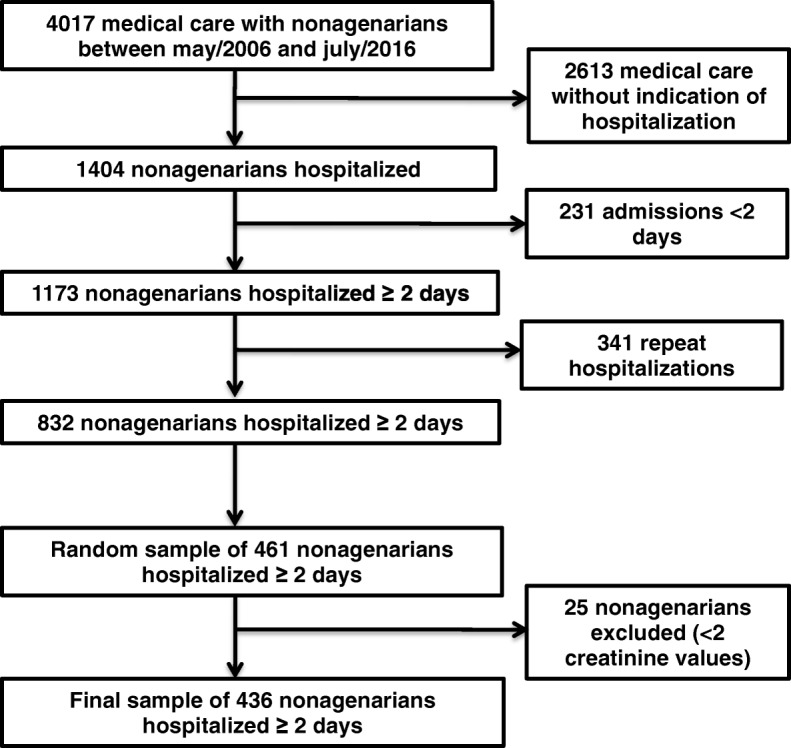
Flow chart demonstrating the process of patient screening and generation of a 436 nonagenarians’ sample

The process of patient screening and generation of a random sample of 436 nonagenarians hospitalized for at least 2 days at a tertiary hospital is demonstrated in Fig. 1. The demographic and clinical characteristics of the final sample are shown in Table 1. The mean age was 93.5 ± 3.3 years and only 25.7% were male. The majority of patients had one or two comorbidities, and the mean age-adjusted Charlson’s comorbidities score was 6 points. It should be noted that 76.4% of the patients required admission to the ICU during the hospital stay; 22% required mechanical ventilation and 31.5% required vasopressors.

**Table 1:** Demographic and clinical characteristics of a random sample of 436 nonagenarians

Variables	n/total	%
Male sex	112/436	25.7%
Age ranges, years		
90 l─l 93	253/436	58.0%
94 I─I 96	104/436	23.9%
≥ 97	79/436	18.1%
Charlson comorbidities scores		
≤ 5	211/436	48.4%
6 I─I 7	149/436	34.2%
≥ 8	76/436	17.4%
Length of hospital stay, weeks		
≤ 1	128/436	29.4%
1 I─I 2	151/436	34.6%
> 3	157/436	36.0%
ICU admission	333/435	76.4%
Time bands of the 1st ICU stay, weeks		
≤ 1	191/331	57.7%
1 I─I 2	70/331	21.1%
> 3	70/331	21.1%
Mechanical ventilation	96/436	22.0%
Vasopressor	137/435	31.5%
AKI	196/436	45.0%
In-hospital mortality	188/436	43.1%

Legend: *ICU* Intensive care unit, *AKI* Acute kidney injury

The incidence of AKI according to KDIGO criteria was 45% (Table 2); approximately half of the AKI episodes were classified as KDIGO stage 1 with the other half almost equally split between KDIGO stages 2 and 3.

**Table 2:** Incidence of AKI and mortality stratified by KDIGO stage, hospital sector, locale and RRT request

AKI	Incidence	95% CI	Mortality	95% CI
KDIGO stage				
KDIGO 1	96/436 (22.0%)	(18.3–25.9)	49/96 (51.0%)	(41.2–60.6)
KDIGO 2	49/436 (11.2%)	(8.3–14.2)	38/49 (77.5%)	(64.8–89.2)
KDIGO 3	51/436 (11.7%)	(8.9–14.4)	44/51 (86.3%)	(76.0–94.9)
Total	196/436 (44.9%)	(40.1–49.3)	131/196 (66.8%)	(60.2–73.6)
Hospital sector				
Ward	23/103 (22.3%)	(15.0–30.4)	18/103 (17.5%)	(10.2–25.2)
ICU	173/333 (51.9%)	(46.5–57.1)	170/333 (51.1%)	(46.1–56.5)
Locale				
Community-acquired	45/196 (23.0%)	(16.9–28.8)	14/45 (31.1%)	(18.2–44.8)
Hospital-acquired	125/196 (63.8%)	(57.1–70.5)	97/125 (77.6%)	(70.2–84.4)
Both	26/196 (13.3%)	(8.8–18.2)	20/26 (76.9%)	(59.1–92.9)
RRT request				
Yes	13/196 (6.6%)	(3.3–10.5)	13/13 (100.0%)	–
No	183/196 (93.4%)	(89.5–96.7)	118/183 (64.5%)	(57.1–72.1)

Legend: *AKI* Acute kidney injury, *CI* Confidence interval, *KDIGO* Kidney Disease Improving Global Outcomes, *ICU* Intensive care unit, *RRT* Renal replacement therapy

Most (63.8%) AKIs were classified as hospital acquired and 24.5% of patients had two or more episodes of AKI during their hospital stay. When we stratified by hospital sector, we found that nonagenarians who were admitted to the ICU had a significantly higher incidence of AKI compared to those who stayed in the wards (51.9% versus 22.3%, *p* < 0.001). Only 13 patients (6.6%) underwent RRT. Of these, 5 received intermittent hemodialysis, 5 continuous venovenous haemodialysis (CVVHD), 1 sustained low-efficiency dialysis (SLED) and 2 patients received SLED and CVVHD. RRT occurred at a median of 2 (IQR 0.5 to 3.0) days after Nephrology consultation. The indications for RRT were hypervolemia in 6 patients, metabolic control in 4 and a mix of hypervolemia and metabolic control in 3. Patients stayed on RRT for a median of 2 (IQR 0.5 to 6.0) days.

Roughly, 11% of the patients used drugs known to be nephrotoxic such as NSAIDs (2.3%) and certain antibiotics (6.0%). Only 26 patients (6%) used iodinated contrast for therapeutic or diagnostic procedures. A significantly higher number, however, used drugs that can affect renal function parameters, such as corticosteroids (27.5%), diuretics (61%) and renin-angiotensin-aldosterone system blockers (38.1%).

**Fig. 2:**
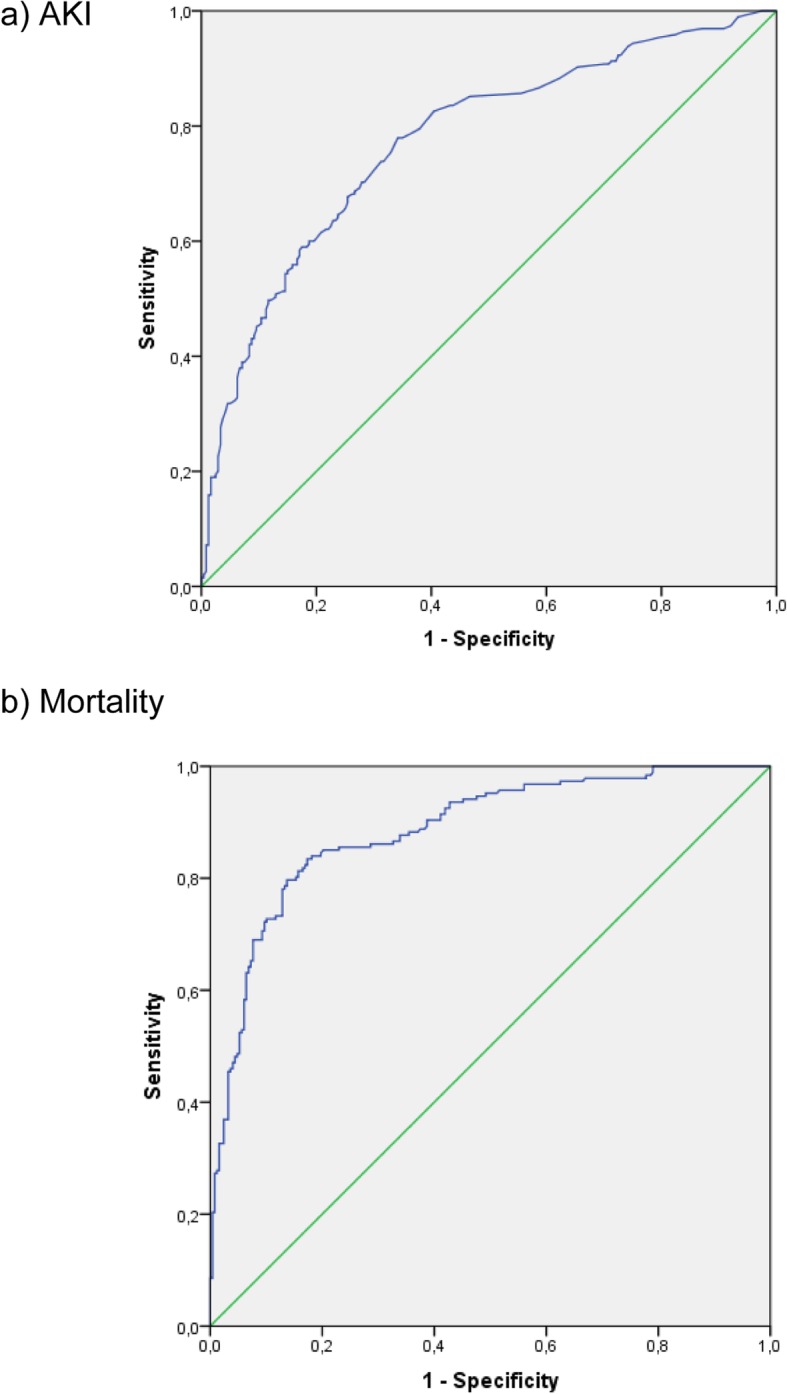
ROC curves for the multivariable logistic regression models for prediction of AKI and mortality. **a** AKI. **b** Mortality

In univariate analyses, eight variables were significantly associated with AKI: male gender, Charlson’s score ≥ 6, length of hospital stay, ICU admission, use of vasopressors, mechanical ventilation, diuretics and corticosteroids (Table 3). These eight variables were then included in a multivariable, stepwise logistic regression model. As shown in Table 3, the independent risk factors for AKI were: length of hospital stay, ICU admission, use of vasopressors and mechanical ventilation. This model had an area under the ROC curve of 0.77 (95% CI: 0.730–0.819, *p* ≤ 0.001) (Fig. 2a).

**Table 3:** Predictors of AKI by univariate and multivariable logistic regression analysis

Variables	AKI		Univariate		Multivariable	
	Yes (*n* = 196)	No (*n* = 240)	OR (CI 95%)	p	OR (CI 95%)	p
Male gender n/total (%)	61/196 (31.1%)	51/240 (21.2%)	1.68 (1.09–2.60)	**0.019**		
Age, years (mean ± SD)	93.4 ± 3.1	93.7 ± 3.6	0.98 (0.93–1.04)	0.480		
Charlson’s score ≥ 6 n/total (%)	113/196 (57.6%)	112/240 (46.7%)	1.56 (1.06–2.28)	**0.023**		
Length of hospital stay, days (mean ± SD)	21 ± 19.4	13 ± 12.1	1.04 (1.02–1.06)	**< 0.001**	1.03 (1.01–1.05)	**0.003**
ICU admission n/total (%)	173/196 (88.3%)	159/240 (66.2%)	3.83 (2.30–6.39)	**< 0.001**	1.83 (1.01–3.35)	**0.048**
Mechanical ventilation n/total (%)	76/196 (38.8%)	20/240 (8.3%)	6.97 (4.06–11.97)	**< 0.001**	2.46 (1.16–5.23)	**0.019**
Vasopressors n/total (%)	103/195 (52.8%)	34/240 (14.2%)	6.78 (4.29–10.73)	**< 0.001**	2.43 (1.29–4.58)	**0.006**
Nephrotoxic antibiotics n/total (%)	13/158 (8.2%)	8/191 (4.2%)	2.05 (0.83–5.08)	0.121		
ACE-i or ARB n/total (%)	62/158 (39.2%)	72/191 (37.7%)	1.04 (0.67–1.60)	0.861		
Diuretics n/total (%)	115/158 (72.8%)	98/191 (51.3%)	2.54 (1.62–3.98)	**< 0.001**		
Corticosteroids n/total (%)	63/158 (39.9%)	33/191 (17.3%)	3.17 (1.94–5.19)	**< 0.001**		
Iodinated contrast	11/195 (5.6%)	15/240 (6.2%)	0.90 (0.80–0.90)	0.790		

Legend: *AKI* Acute kidney injury, *OR* Odds ratio, *CI* Confidence interval, *SD* Standard deviation, *ICU* Intensive care unit, *ACE-i* Inhibitor of angiotensin-converting-enzyme, *ARB* Angiotensin II receptors blockers. The following variables entered the multivariable logistic regression model: male gender, Charlson’s score ≥ 6, length of hospital stay, ICU admission, mechanical ventilation, vasopresssors, diuretics and corticosteroids. The last column only shows the ones that remained statistically significant at the last step of the multivariable model

Bold entries are variables with p values < 0.10 on univariate analyses were selected for inclusion in stepwise backward multivariable logistic regression models and variables with p values < 0.05 in final analyses were considered statistically significant

Overall, hospital mortality was 43.1%. Mortality was significantly higher in the AKI than in the no AKI group (66.8% versus 23.8%, *p* < 0.001); interestingly, when stratified by locale, only hospital-acquired AKI was significantly associated with increased mortality. There was a positive correlation between number of episodes and severity of AKI with mortality. The association between AKI and mortality was stronger in the ICU than in the wards (Table 2). In the ICU, mortality was significantly higher in the AKI than in the no AKI group (71.7% versus 28.8%, p < 0.001). For nonagenarians hospitalized exclusively in the wards, there was only a trend towards higher mortality in the AKI group (30.4% versus 13.8% in the no AKI group; *p* = 0.063). In patients that underwent RRT, mortality reached 100%. The group that underwent RRT was comprised of critically ill patients: 100% were in the ICU and on vasopressors; 76.9% were on mechanical ventilation.

In univariate logistic regression analyses, the following variables were associated with mortality: Charlson’s score ≥ 6, length of hospital stay, ICU admission, use of vasopressors, mechanical ventilation, AKI and RRT. These variables were included in a multivariable, stepwise backward logistic regression analysis. Although age did not show a statistically significant association with mortality in univariate analysis, we decided to include it in the multivariable model given the relevance of age to any analysis of mortality. In the final model, age, Charlson’s score ≥ 6, use of vasopressors, mechanical ventilation and AKI (at all stages) remained independently associated with mortality (Table 4). The area under the ROC curve for this model was 0.89 (95% CI: 0.85–0.92, *p* < 0.001) (Fig. 2b). In the multivariable analysis, performance of RRT was not independently associated with mortality. To further explore this issue, we created a random sample of controls (2 controls for each RRT case) for the RRT group paired by clinical severity. In this control group, all patients had AKI, were in the ICU and on vasopressors and 76.9% were on mechanical ventilation. Mortality in this non-RRT control group was 96%, which was not significantly different than that observed in the RRT group (*p* = 1.0) {Additional file 1: Table S1).

**Table 4:** Predictors of mortality by univariate and multivariable logistic regression analysis

Variables	Mortality		Univariate		Multivariable	
	Yes (*n* = 188)	No (*n* = 248)	OR (CI 95%)	p	OR (CI 95%)	p
Male gender n/total (%)	56/188 (29.8%)	56/248 (22.6%)	1.45 (0.94–2.24)	0.89		
Age, years (mean ± SD)	93.9 ± 3.4	93.3 ± 3.4	1.05 (0.99–1.11)	0.96	1.12 (1.04–1.21)	**0.002**
Charlson’s score ≥ 6 n/total (%)	144/188 (76.6%)	111/248 (44.8%)	1.90 (1.30–2.80)	**0.01**	1.69 (1.01–2.83)	**0.049**
Length of hospital stay, days (mean ± SD)	21.2 ± 20.3	13.1 ± 11.2	1.04 (1.02–1.05)	**< 0.001**		
ICU admission n/total (%)	170/188 (90.4%)	162/248 (65.3%)	5.01 (2.89–8.70)	**< 0.001**		
Mechanical ventilation n/total (%)	87/188 (46.3%)	9/248 (3.6%)	22.87 (11.08–47.21)	**< 0.001**	5.29 (2.24–12.46)	**< 0.001**
Vasopressors n/total (%)	116/187 (62.0%)	21/248 (8.5%)	17.66 (10.33–30.17)	**< 0.001**	6.82 (3.55–13.11)	**< 0.001**
AKI all cases	131/188 (69.7%)	65/248 (26.2%)	6.47 (4.25–9.85)	**< 0.001**		
AKI KDIGO 1	49/188 (26.1%)	47/248 (18.9%)	3.35 (2.03–5.51)	**< 0.001**	2.01 (1.09–3.72)	**0.025**
AKI KDIGO 2	38/188 (20.2%)	11/248 (4.4%)	11.09 (5.32–23.11)	**< 0.001**	4.95 (2.05–11.94)	**< 0.001**
AKI KDIGO 3	44/188 (23.4%)	7/248 (2.8%)	20.18 (8.61–47.27)	**< 0.001**	6.52 (2.37–17.98)	**< 0.001**
Community AKI	14/188 (7.4%)	31/248 (12.5%)	1.45 (0.72–2.91)	0.297		
Hospital AKI	97/188 (51.6%)	28/248 (11.3%)	11.12 (6.65–18.61)	**< 0.001**		
Community and Hospital AKI	20/188 (10.6%)	6/248 (2.4%)	10.70 (4.10–27.94)	**< 0.001**		
RRT	13/188 (6.9%)	0/248 (0.0%)	(−)	**< 0,001**		

Legend: *RRT* Renal Replacement Therapy, *OR* Odds ratio, *CI* Confidence interval, *SD* Standard deviation, *ICU* Intensive care unit, *AKI* Acute kidney injury, *KDIGO* Kidney Disease Improving Global Outcomes. Note: the following variables entered the multivariable logistic regression model: age, Charlson’s score ≥ 6, length of hospital stay, ICU admission, mechanical ventilation, vasopresssors, AKI stage and RRT. The last column only shows the ones that remained statistically significant at the last step of the multivariable model

Bold entries are variables with p values < 0.10 on univariate analyses were selected for inclusion in stepwise backward multivariable logistic regression models and variables with p values < 0.05 in final analyses were considered statistically significant

## Discussion

We analyzed a random sample of 436 nonagenarians hospitalized for at least 2 days at a tertiary hospital and found that the incidence of AKI by KDIGO criteria was 45%. AKI was more common in those with a longer hospital stay, need for ICU, vasopressors or mechanical ventilation. More importantly, all stages of AKI were independently associated with mortality, which reached 100% in those undergoing RRT. However, performance of RRT was not a predictor of mortality. Mortality in the RRT group was also not significantly different than that observed in the controls matched by severity of illness (ICU, vasopressors and mechanical ventilation).

When comparing the incidence of AKI across different studies, some aspects need to be considered, such as the diagnostic criterion adopted and the population studied. Using the same dataset, Lassnigg et al. demonstrated how AKI might vary from a rare to a very common condition by simply applying different diagnostic criteria [11]. Regarding the population, it is also quite clear that the incidence of AKI will be higher in studies of critically ill patients than in studies of patients in the wards. Herein, we applied the most sensitive AKI criteria available – the KDIGO criteria – to a very high-risk group of individuals: gravelly ill nonagenarians, more than ¾ of whom required ICU care at some point during hospitalization. The incidence of AKI was 45% (KDIGO stage 1 or greater). We did not find other studies reporting the incidence of AKI according to the KDIGO criteria in critically ill nonagenarians to allow an adequate comparison to our data.

Chao et al. reported an incidence of AKI of 5.3% in 283 nonagenarians admitted to a tertiary center in Taiwan [9]. However, they used the RIFLE criteria, which is less sensitive than KDIGO, and only 23% of their patients required ICU admission [12]. Wen et al. reported an incidence of AKI of 14.8% according to AKIN criteria in very elderly individuals, with a mean age of 88 years old. However, the authors did not provide detailed information regarding the clinical severity of their patients [8]. An Italian multicenter, prospective cohort study investigating 576 critically individuals, mean age 66 years old, detected an incidence of AKI according to the RIFLE criteria of 65.8% [7]. In our study, the incidence of AKI was 51.2% in patients admitted to the ICU and 22.3% in those who did not require ICU admission.

Age has been described as a risk factor for AKI in several studies [3, 7, 8, 13]. Patients over 80 years old are 5 times more likely to develop AKI than younger counterparts [14]. A recent Irish cohort study showed that the adjusted incidence rate ratios for patients > 80 years of age were > 11-fold higher than for patients 18–39 years of age [15]. The loss of renal reserve due to renal senescence, multiple comorbidities and polypharmacy may account for these findings [4, 6]. In our study, however, which involved only nonagenarians, age was not a risk factor for AKI.

The independent risk factors for AKI in our cohort of nonagenarians were length of hospital stay, ICU admission, vasopressor use and mechanical ventilation. All of these variables are related to disease severity. In other AKI studies, including those in the elderly population, multiple organ dysfunction syndrome (MODS) was associated with AKI [9, 16].

Mortality in the elderly with AKI varies in the literature from 23% [17] to 54% [13] according to the mean age of the patients, severity of illness and criteria used to define AKI. In our series, almost 70% of the nonagenarians with AKI died. There was a direct association between mortality and KDIGO stage and the number of AKI episodes. The chance of death more than doubled for each elevation of the KDIGO stage, even after controlling for variables such as: age, length of hospital stay, Charlson comorbidity score, ICU admission, vasopressor use, mechanical ventilation and need for RRT. Nonagenarians with AKI KDIGO stage 3 had a 6-fold higher chance of dying when compared to those who did not develop AKI. Even AKI stage 1 was associated with mortality, confirming what has been described in younger patients with AKI [18].

Data from the University of Pittsburgh (15,000 patients) suggest that approximately one third of AKI cases are acquired in the community, a fact that should be more common in developing countries in coming years [19]. Community-acquired AKI was not associated with mortality in our study. This contrasts with data from Chia-Ter Chao et al. who found higher in-hospital mortality in nonagenarians admitted with AKI [9]. However, in that study, the AKI criterion was different: an absolute increase in SCr of ≥0.5 mg/dl on hospital admission when compared to previous outpatient values. We diagnosed AKI according to KDIGO criteria and the lowest SCr of hospitalization was defined as the baseline. Another cohort using RIFLE criteria found a better prognosis for community-acquired AKI when compared to hospital-acquired AKI, in agreement with our results [20].

The in hospital-mortality of 13 nonagenarians who required RRT was 100%. However, all 13 patients in the RRT group were in the ICU and had circulatory shock requiring vasopressors; most had respiratory failure requiring mechanical ventilation. Our hypothesis is that the performance of RRT was not able to modify the fatal course of these 13 critically ill nonagenarians, but it was also not responsible for their death. This was suggested by two separate analyses. First, RRT was not an independent predictor of mortality in multivariable logistic regression. Second, we created a control group of 26 nonagenarians with AKI obtained by proportional random sampling that had the same clinical severity but did not undergo RRT. Mortality in this control group was 96%, which was not statistically different than that observed in the RRT group (*p* = 1.0), thus not showing evidence for a direct association between RRT and mortality. A recent Brazilian study, using propensity-score matching analysis, also found no impact of RRT on mortality in individuals over 70 years old with AKI [21].

The dilemma about the use of RRT in elderly patients has been gaining ethical and legal force in recent years [22]. The technical possibility of healing, albeit remote, and the prolongation of life by artificial means offered by health technology, often clashes with the primary goal of minimizing suffering and maximizing quality of the remaining life. The technological imperative in health, in this sense, has morally influenced the prescription of RRT by nephrologists. However, some studies in adults have also warned to increased risk of death when RRT is prescribed in patients with SCr < 3.8 mg/d [23]. Our data suggests that performance of RRT in nonagenarian patients with AKI, in mechanical ventilation, and with circulatory shock does not save lives.

This paper has as main limitations: a) retrospective design, which limited data collection to what was available in electronic medical records. For example, we did not have consistent and reliable data regarding diuresis. Therefore, our incidence of AKI is derived from creatinine data only. This is rather common in studies that involve patients outside the ICU, where intake / output data is not rigorously registered. We also did not have data on the usual illness severity scores. Nevertheless, we were able to use the Charlson’s comorbidity index, ICU admission, and need for vasopressors and mechanical ventilation to infer clinical severity; b) A few aspects of our sample selection strategy may have contributed to drawing a sicker group of patients: the need for a hospital length of stay of at least 2 days, the need for at least 2 serum creatinine values and the choice of a patients’ last hospitalization when multiple hospitalizations occurred. The first two requirements had to do with establishing the diagnosis of AKI. Regarding multiple hospitalizations, choosing any admission other than the last equaled choosing a patient that certainly did not die. Although choosing the last admission increased the probability that the patient died during that admission, it would not amount to a certainty, as patients in our health system are often admitted to different hospitals. Therefore, the last admission to our hospital does not necessarily mean the last admission of the patients’ life; c) the fact that it was conducted in a single center, which limits the external validity. However, this study was conducted in a typical tertiary hospital in Brazil. Therefore, we consider unlikely that the results found herein are due to the peculiar characteristics of the patients and the care provided at our center. Moreover, we evaluated almost 500 patients, the largest cohort of nonagenarians with AKI in the literature; d) our regression coeficients and C-statistics might be inflated due to overfitting to our particular cohort; e) data on RRT are based on a sample of only 13 critically ill patients, thus limiting conclusions. Our data do not allow us to extrapolate about the mortality in RRT of nonagenarians who don’t have the triad: AKI, circulatory shock and respiratory failure requiring mechanical ventilation.

## Conclusion

AKI occurred frequently in hospitalized nonagenarians and was independently associated with markers of illness severity, such as length of hospital stay, ICU admission, vasopressor use and mechanical ventilation. Even mild episodes of AKI had an impact on mortality. Severe AKI increased mortality more than six-fold and RRT did not improve the prognosis of critically ill nonagenarians with AKI. Additional research is required in nonagenarians with AKI, especially in those requiring RRT, to further inform clinicians on the utility / futility of RRT in this population.

## Additional file

**Additional file 1 **: **Table S1.** Mortality comparison between the group of nonagenarians who underwent RRT and a non-RRT control group obtained by proportional random sampling matched by variables related to illness severity.

## Data Availability

The datasets used and/or analysed during the current study are available from the corresponding author on reasonable request.

## Abbreviations

AKI: Acute kidney injury
AKIN: Acute Kidney Injury Network
BEST: Beginning and Ending Supportive Therapy for the Kidney
CVVHD: Continuous venovenous haemodialysis
ICU: Intensive care unit
IQR: Interquartile range
KDIGO: Kidney Disease Improving Global Outcomes
MODS: Multiple organ dysfunction syndrome
MV: Mechanical ventilation
RIFLE: Risk, Injury, Failure, Loss of kidney function, and End-stage kidney disease
ROC: Receiver operating characteristic
RRT: Renal replacement therapy
SCr: Serum creatinine
SLED: Sustained low-efficiency dialysis
SPSS: Statistical package for social sciences
