# Supplementary material for: Incidence, predictors and prognosis of acute kidney injury in nonagenarians: an in-hospital cohort study
Source: BMC Nephrol. 2020 Jan 30;21:34. doi: 10.1186/s12882-020-1698-y (PMC6993395; doi:10.1186/s12882-020-1698-y)
Supplement: Supplementary file 1 — Additional file 1 : Table S1. Mortality comparison between the group of nonagenarians who underwent RRT and a non-RRT control group obtained by proportional random sampling matched by variables related to illness severity. [file 12882_2020_1698_MOESM1_ESM.docx]

Additional file 1: **Table S1.** Mortality comparison between the group of nonagenarians who underwent RRT and a non-RRT control group obtained by proportional random sampling matched by variables related to illness severity.

| Variables | RRT | | p |
| --- | --- | --- | --- |
|  | Yes  (n=13) | No  (n=26) |  |
| Male gender  n/total (%) | 5/13  (38.5%) | 9/26  (34.6%) | 1.0 |
| Age  (mean ± SD) | 93.31 ± 2.01 | 93.0 ± 3.63 | 0.78 |
| Charlson’s score ≥6  n/total (%) | 8/13  (61.5%) | 16/26  (61.5%) | 1.0 |
| Length of hospital  stay, days  (mean±SD) | 16.08 ± 9.98 | 22.50 ± 16.02 | 0.20 |
| ICU admission  n/total (%) | 13/13  (100%) | 26/26  (100%) | 1.0 |
| Mechanical ventilation  n/total (%) | 10/13  (76.9%) | 20/26  (76.9%) | 1.0 |
| Vasopressors  n/total (%) | 13/13  (100%) | 26/26  (100%) | 1.0 |
| Mortality  n/total (%) | 13/13  (100%) | 25/26  (96.1%) | 1.0 |

_Legend: RRT = Renal replacement therapy; SD = standard deviation; ICU = intensive care unit_
